# Coevolutionary Patterns in SOS1 and NHX1: Insights into Plant Ion Homeostasis Proteins

**DOI:** 10.3390/ijms26199276

**Published:** 2025-09-23

**Authors:** Mario X. Ruiz-González, Antonio Vélez-Mejía, Oscar Vicente

**Affiliations:** Institute for the Conservation and Improvement of Valencian Agrodiversity (COMAV), Universitat Politècnica de València, Camino de Vera s/n, 46022 Valencia, Spain; avelmej@posgrado.upv.es (A.V.-M.); ovicente@upvnet.upv.es (O.V.)

**Keywords:** coevolution, NHX1, SOS1, protein function, protein interaction, protein topology, salt tolerance

## Abstract

The SOS (salt overly sensitive) hypersensitivity pathway is a key mechanism for maintaining ion homeostasis at the cellular level and conferring plant resistance and tolerance to salt stress. Its components interact directly and indirectly with various proteins and regulatory mechanisms. We conducted the first coevolutionary analysis of two key proteins of the SOS interaction network (SOS1 and NHX1) across a broad taxonomic range of plant species, including halophytes and glycophytes. Due to sequence availability, our analyses primarily support intramolecular coevolutionary patterns, with preliminary indications of possible intermolecular associations. We assessed the functional and topological relevance of coevolving sites. Six coevolving amino acid pairs were identified in SOS1, and two in NHX1. Except for two residues in SOS1, all sites were associated with functionally and topologically conserved positions. In SOS1, the most relevant coevolving pairs were located in the cytoplasmic domain, which controls the activity of the Na^+^/H^+^ antiporter and plays a critical role in maintaining cellular Na^+^ homeostasis, whereas in NHX1, they were in the transmembrane domain. Our findings reveal previously unexplored molecular relationships in these critical ion homeostasis proteins. Understanding these interactions, which have significant implications for biotechnology and sustainable agriculture, can aid crop improvement and enhance agricultural sustainability under saline conditions.

## 1. Introduction

The effects of climate change are widespread and affect all regions of the planet with varying intensity, generating diverse and complex impacts on biodiversity, and putting at risk the survival of species, human health, and the economy [1,2]. Amongst the most drastic effects of climate change is soil salinisation, which can lead to a loss of biodiversity and crop yields, and affects at least 75 countries [3]. The increase in salt concentration in soil causes an ionic imbalance in the plant, which affects its growth and development by altering cellular metabolism, photosynthesis, and root architecture, ultimately leading to plant death [4].

Plant communities have adapted to harsh environmental conditions through a range of tolerance mechanisms at different scales: structural and functional modifications at the individual level—such as changes in stomatal behaviour and downregulation of photosynthesis—that minimise water loss and limit damage under stress [5]; at the biochemical and molecular scale, there are three main mechanisms of salt tolerance, responding to the most significant adverse effects of salt stress: osmotic stress, oxidative stress, and ion toxicity [6,7,8,9,10]. That is, plants accumulate osmoprotective compounds, such as glycine betaine, proline or soluble sugars, to maintain cellular osmotic balance, help stabilise proteins and cellular structures, and also maintain turgor without interfering with metabolism [9,11]. Tolerance to oxidative stress involves mobilising enzymatic and non-enzymatic antioxidant defences to degrade reactive oxygen species (ROS) and mitigate the oxidative damage caused by elevated ROS levels. These reactions are catalysed by various antioxidant enzymes, including ascorbate peroxidase (APX), glutathione S-transferase (GST), glutathione reductase (GR), superoxide dismutase (SOD), and catalases, whereas common antioxidant metabolites include phenolic compounds, carotenoids, vitamins C and E, or reduced glutathione [9,12]. The third mechanism involves regulating ion transport (e.g., exclusion of toxic ions from photosynthetic tissues, or their sequestration in the vacuoles) and the maintenance of ionic homeostasis [9].

**Figure 1:**
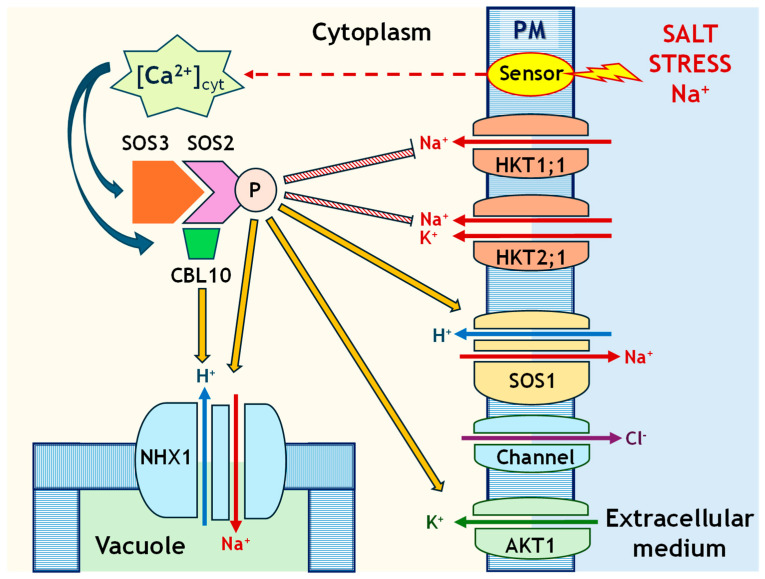
SOS signalling pathway (salt overly sensitive). Salt stress is perceived at the membrane level (PM, plasmatic membrane), leading to an increase in cytosolic Ca^2+^ concentration ([Ca^2+^]_(cyt)_), which is detected by the calcium sensor SOS3. SOS3 both recruits the SOS1 antiporter to the membrane and forms the SOS3/SOS2 kinase complex, which, upon phosphorylation, activates the membrane-bound SOS1 antiporter. SOS2 can also regulate the activity of the NHX1 antiporter by promoting Na^+^ efflux in exchange for H^+^. AKT1, *Arabidopsis* K^+^ Transporter 1.

In contrast to glycophytes, halophytes can tolerate salt stress by transporting toxic ions such as Na^+^ outside the cells or storing them inside vacuoles or specific tissues to maintain cellular homeostasis [13]. Comparing the stress responses of halophytes and glycophytes enables us to study the mechanisms of adaptation and tolerance to salt stress and identify genes associated with salinity tolerance, with potential biotechnological applications. The ion exclusion mechanism starts when Na^+^ ions enter the cell and salt stress is perceived at the membrane level, which rapidly increases cytosolic Ca^2+^ levels (Figure 1). This rise acts as a signal that activates the SOS (salt overly sensitive) signalling pathway. The SOS3 protein (also known as CBL4, calcineurin B-like protein 4), which functions as a calcium sensor, binds to Ca^2+^, undergoing a conformational change that allows it to interact with the kinase SOS2 (CIPK24, CBL-interacting protein kinase 24). Subsequently, the SOS3–SOS2 complex is formed, which activates the kinase. Then, activated SOS2 phosphorylates and activates the SOS1 antiporter located in the plasma membrane. SOS1 is a Na^+^/H^+^ exchanger that uses the proton gradient to expel excess Na^+^ from the cytoplasm to the cell exterior, in exchange for H^+^ ions [14]. At the same time, it promotes the degradation of the high-affinity K^+^ transporter 1, HKT1 [15]. HKT1 passively transports Na^+^, while anion channels similarly mediate Cl^−^ transport (Figure 1). Two subfamilies of the HKT1 transporter have been identified. Subfamily 1 (HKT1;1), found in *Arabidopsis thaliana*, functions as a low-affinity, Na^+^-selective transporter, whereas subfamily 2 (HKT2;1), present in *Triticum aestivum*, acts as a Na^+^/K^+^ symporter [9]. Both forms of HKT1 are localised to the plasma membrane (PM), although typically only one form is present in a given species (Figure 1). SOS2 can also regulate the activity of the NHX1 (Na^+^/H^+^ exchanger 1) antiporter by promoting Na^+^ efflux from the cytoplasm into the vacuole and supporting Na^+^ loading into the xylem, through its interaction with calcineurin B-like protein 10, CBL10 [16].

Ion transport proteins are encoded by genes involved in salinity tolerance and possess a specific structural composition that, despite being highly conserved, can vary among different plant species [17]. These variations arise from the accumulation of mutations across different evolutionary lineages over time. A fundamental aspect of molecular evolution is the impact that a mutation can have on protein coding. Nucleotide substitutions may or may not change the corresponding amino acid sequence. These are referred to as non-synonymous or synonymous mutations, respectively [18]. Only non-synonymous mutations lead to changes that can affect a protein’s structure and function. Thus, in addition to expression levels, changes in protein structure can result in differences in the efficiency of salt stress tolerance. Molecular changes in genes with key biological functions are often subjected to strong selective pressures [19], which can give rise to complex evolutionary dynamics, such as coevolutionary processes [20].

Coevolution is a foundational concept in biological evolution. It refers to the process by which two or more organisms exert mutual and synchronised selective pressures (on a geological timescale), resulting in specific reciprocal adaptations. The concept of coevolution was first introduced by Ehrlich and Raven [21]. Furthermore, coevolution implies that the selective pressures exerted by one species on another can lead to complex evolutionary dynamics, such as evolutionary “arms races.” This idea was articulated by Leigh Van Valen [22] as the Red Queen hypothesis, which describes how species must continually adapt not only to the physical environment but also to the species with which they interact, to maintain their relative biological fitness. In addition, selective pressures can be geographically localised, giving rise to spatially variable coevolutionary dynamics [23]. In the context of plants, such geographically driven selection may stem from variations in soil salinity.

Coevolutionary processes were described initially at the macroevolutionary level, occurring between species. However, coevolutionary dynamics also involve genetic changes within the interacting species’ lineages, giving rise to a different form of coevolution, molecular coevolution, in which evolutionary change occurs between interacting molecules within a single lineage, such as receptor proteins and their ligands [20,24]. In this context, a mutation in one gene influences the selective pressure acting on another gene due to the interaction of their protein products, and this effect is reciprocal. At the molecular level, a mutation can also produce changes in the three-dimensional conformation of a molecule, thereby affecting its physicochemical properties and function [25]. It is essential to distinguish between the two types of coevolutionary dynamics that occur at the molecular level [26]: intermolecular coevolution, as previously described, occurs between two genes whose products interact within an organism; and intramolecular coevolution, which takes place within a single molecule, driven by strong selective pressures to preserve specific properties of that molecule. A consideration in the study of intermolecular coevolution is the phenomenon of Evolutionary Rate Covariation (ERC), whereby two proteins exhibit similar evolutionary patterns over time without necessarily being physically linked or directly interacting [27]. This occurs because functionally related proteins may experience similar evolutionary pressures. For example, if one protein is highly conserved (i.e., under strong purifying selection, that is, the process by which natural selection eliminates harmful genetic variants from a population), its functional partners may be similarly constrained [28]. Alternatively, if an adaptive change arises due to a shift in a metabolic pathway, multiple proteins within that pathway may evolve simultaneously [29]. While intermolecular coevolution and ERC are interrelated, they are not equivalent. ERC refers specifically to the correlation in evolutionary rates between pairs of proteins over evolutionary time.

Within a protein, changes at most sites can affect its structure and function. However, some sites may hold greater coevolutionary relevance than others [30]. Specific mutations that drastically alter protein structure and/or function can be compensated by additional mutations occurring elsewhere in the same protein, such that only when both changes are present is the protein’s functionality preserved [31]. Indeed, amino acid substitutions can be context-dependent, helping avoid protein stability disruptions [32]. Since most mutations result in loss of function [33], compensatory changes may also occur in the interacting partner protein, thereby preserving overall function. Some amino acid substitutions can lead to deleterious or mildly deleterious effects, which may be offset by specific compensatory changes at other amino acid positions (e.g., [34,35]). Coevolutionary analysis of amino acid residues within proteins offers a powerful tool for uncovering residues essential to optimising molecular responses to salt stress [32,36]. Moreover, studying coevolving amino acids is important for genetic improvement, as it facilitates the identification of mutations that enhance protein performance under specific conditions, such as salinity [37]. Specific amino acids have been shown to be critical for the stability and function of NHX1 [38]. However, potential intramolecular coevolutionary interactions in genes involved in ion homeostasis remain unexplored.

Molecular coevolution is often driven by selective pressures acting on genes whose protein products interact directly. However, physical interaction is not a prerequisite for molecular coevolution. Genes whose products are functionally associated—such as those coactivated in response to sodium ion stress—may evolve in a correlated manner even without direct physical interaction. In such cases, coordinated selective pressures can drive functional coupling between proteins acting within the same physiological context. Mutations in one sequence may require compensatory changes in the other to maintain cellular homeostasis, giving rise to coevolutionary dynamics between amino acid residues of both proteins. This may be the case for NHX1 and SOS1, which are both activated by sodium stress: NHX1 facilitates sodium sequestration into the vacuole, whereas SOS1 exports sodium out of the cell [39].

Given the crucial role of ion homeostasis in plants [40], we hypothesised that pairs of coevolving amino acids exist both within and between proteins involved in ion transport, with these residues playing essential roles in the function and/or topology of such proteins. To address this issue, we evaluated the availability of sequence data necessary for conducting coevolutionary analyses within the protein network responsible for ion homeostasis in plants. We then examined potential intramolecular and intermolecular coevolutionary patterns in the NHX1 and SOS1 genes. Finally, we performed computational analyses to determine whether changes at coevolving amino acid sites could influence the function and structural stability of SOS1 and NHX1.

## 2. Results

### 2.1. Data Acquisition and Sequence Curation

The SOS3 (CBL4) interaction network revealed 16 genes in *Arabidopsis*, nine showing direct interactions and seven forming a second layer of indirect associations. Raw protein sequence data were retrieved for each of these from UniProt (Table 1). As the primary goal at this stage was to perform coevolutionary analyses, a large number of sequences is essential to ensure statistical robustness. Furthermore, sufficient evolutionary diversity among the sequences is required to generate informative results. Although the initial study design aimed to include SOS3-related proteins, only SOS1 and NHX1 met these criteria and were suitable for further analysis (Table 1). For SOS1, we selected 36 species belonging to 17 taxonomic families and 11 orders (Asterales, Brassicales, Caryophyllales, Cucurbitales, Fabales, Malpighiales, Malvales, Poales, Sapindanales, Solanales, and Vitales; Supplementary Materials, Table S1). For NHX1, we selected 62 species belonging to 22 families and 17 orders (Asparagales, Asterales, Brassicales, Caryophyllales, Fabales, Gentaniales, Lamiales, Malpighiales, Malvales, Poales, Proteales, Ranunculales, Rosales, Sapindanales, Saxifragales, Solanales, and Vitales; Supplementary Materials, Table S2). These selections cover a broad taxonomic range across angiosperms, representing both monocot and dicot lineages.

### 2.2. Intra- and Intermolecular Coevolutionary Analysis

**Figure 2:**
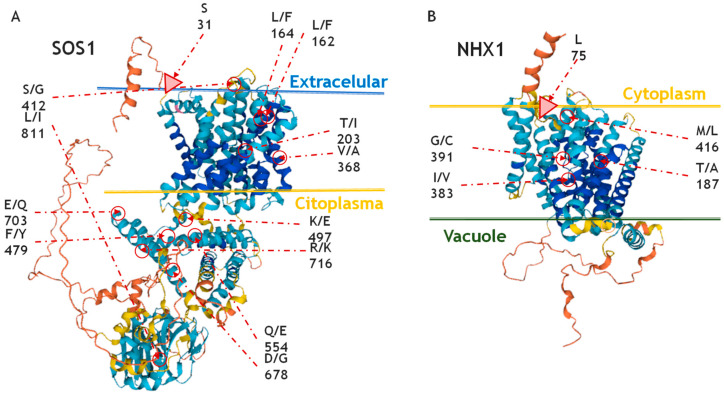
Pairs of residues in coevolution within *Arabidopsis thaliana* SOS1 and NHX1 proteins. (**A**) Coevolving site pairs in SOS1: CAPS detected six pairs of coevolving sites: 162–164, 203–368, 412–811, 479–703, 497–716, and 554–678. Lines separate the cytoplasmic, transmembrane, and vacuolar regions. (**B**) Coevolving site pairs in NHX1: CAPS identified two pairs of coevolving sites: 187–416 and 383–391. In the figure, each site is labelled with its position in the *A. thaliana* protein sequence and annotated with two amino acids as aa1/aa2, where aa1–aa1 denotes the most frequent co-occurring amino acid pair, and aa2–aa2 indicates an alternative co-mutated pair observed in the alignment. Arrows with big triangle heads point to each of the residues potentially involved in intermolecular coevolution. The 3D models correspond to *A. thaliana* SOS1 (Q9LKW9) and NHX1 (Q68KI4) sequences predicted by AlphaFold (https://alphafold.ebi.ac.uk, accessed 4 February 2025).

The intramolecular analysis found six pairs of sites in coevolution in SOS1 and two pairs in NHX1 (Table 2). In SOS1, the first pair corresponded to positions 162 and 164 in AtSOS1, occurring in 86.49% of the species analysed. Both positions were leucines, except in the *Aegilops* sp.—*Triticum* sp. group, where they were replaced by phenylalanines (Table 2). The second pair was located at positions 203 and 368, corresponding to threonine and valine, respectively, and occurred in 94.60% of the species, except in *Bassia* sp. and *Salicornia* sp. (Caryophyllales), where they were replaced by isoleucine and alanine. The third pair involved positions 412 and 811, which were serine and leucine, respectively, and occurred in 94.60% of the sequences, but were substituted by glycine and isoleucine in *Ricinus* sp. (Malpighiales) and *Glycine* sp. (Fabales). The fourth pair was found at positions 479 and 703 (phenylalanine and glutamate), with 94.60% occurrence, except in *Fagopyrum* sp. (Caryophyllales), where they changed to tyrosine and glutamine. The fifth pair corresponded to positions 497 and 716 (lysine and arginine), also found in 94.60% of species, but replaced by glutamate and lysine in *Arabidopsis* sp. and *Eutrema* sp. (Brassicales). A similar pattern was observed in the sixth pair (positions 554 and 678), composed of glutamine and aspartate, which were substituted by glutamate and glycine in the same genera. Three of these pairs of sites, along with a seventh individual site, were in the cytoplasmic region; the second residue of the fourth pair was found in the extracellular region; and two pairs were situated within the transmembrane domain (Figure 2A). In NHX1, both pairs of coevolving sites had an occurrence of 96.8% and corresponded to positions T187–M416 and I401–G391, respectively. These pairs were conserved across all species analysed, except in *Paeonia* (Saxifragales) and *Gentiana* (Gentianales), where they were replaced by A187–L416 and V401–C391, respectively (Table 2, Figure 2B).

The intermolecular coevolution analysis between SOS1 and NHX1, performed on the nine species for which sequences of both proteins were available, revealed seven pairs of potential coevolving sites between the two genes, with a *p*-value of 0.001 (Table 3). Among these, only one pair showed a bootstrap value greater than 0.95, corresponding to two amino acids located within the transmembrane regions of both proteins. Additionally, seven amino acid positions in SOS1 may be coevolving with only three amino acid positions in NHX1 (69, 75, and 456).

### 2.3. Effects of Amino Acid Residues on SOS1 and NHX1 Function

The mutation of each amino acid site in SOS1 and NHX1 to all possible alternative residues allowed us to estimate in silico the intolerance of each position to substitution and to quantify the total number of residues within defined intolerance ranges (Table 4). In SOS1, over 67% of residues (769) showed a substitution intolerance score above 0.75, half of which had a predicted loss-of-function probability of 0.95. This score indicates that approximately 33% of SOS1 residues are highly sensitive to any non-synonymous mutation, potentially compromising protein function. In contrast, 5% of the residues had an intolerance score of 0.00, suggesting they were unaffected by substitution. Similarly, in NHX1, 77.9% of residues were predicted to have a strong functional impact when mutated, with 50% of these positions associated with a loss-of-function score of 0.95 (Table 4).

**Figure 3:**
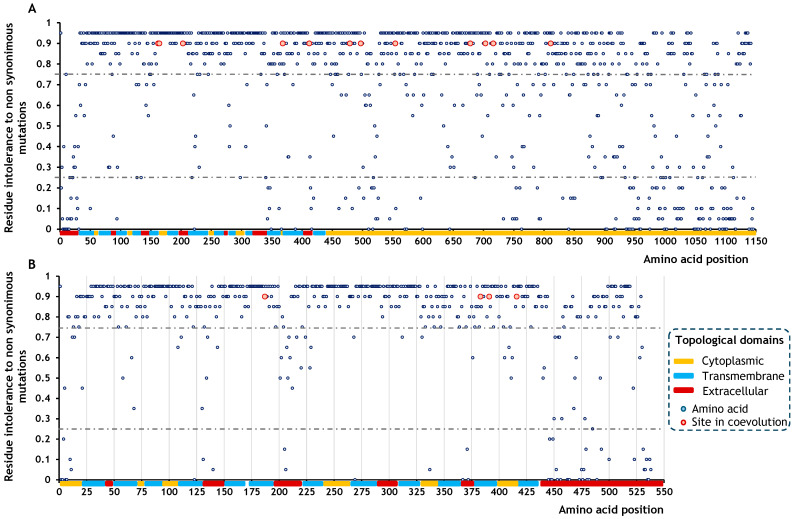
Map of predicted loss-of-function effects caused by non-synonymous mutations in SOS1 and NHX1. (**A**) SOS1; (**B**) NHX1. Mutation intolerance was predicted using the SIFT algorithm (https://sift.bii.a-star.edu.sg/, accessed 2 June 2025). Dashed lines indicate intolerance thresholds, where values below 0.25 are expected to have minimal impact on function, and values above 0.75 are associated with protein inactivation or loss of function. Functional domains are colour-coded: C (orange) corresponds to the cytoplasmic topological domain; E (dark red) represents the extracellular domain in SOS1 or the vacuolar domain in NHX1; and H (blue) marks the transmembrane α-helical segments. Coevolving sites are highlighted as enlarged red circles.

This information enabled the generation of site-specific mutation intolerance maps, allowing visualisation of the functional importance of individual amino acids and coevolving residue pairs. The six coevolving site pairs identified in SOS1 were distributed along the sequence but consistently involved residues with intolerance values of 0.90 (Figure 3A). Most of these pairs were located within the cytoplasmic topological domain, with only one pair found in a transmembrane segment and another in the extracellular domain. In NHX1, the four residues comprising the two coevolving site pairs also showed a predicted loss-of-function probability of 0.90 (Figure 3B). All these residues were located within transmembrane regions.

### 2.4. Effects of Amino Acid Residues on SOS1 and NHX1 Topology

#### 2.4.1. Effects of Amino Acid Residues on SOS1 Topology

**Figure 4:**
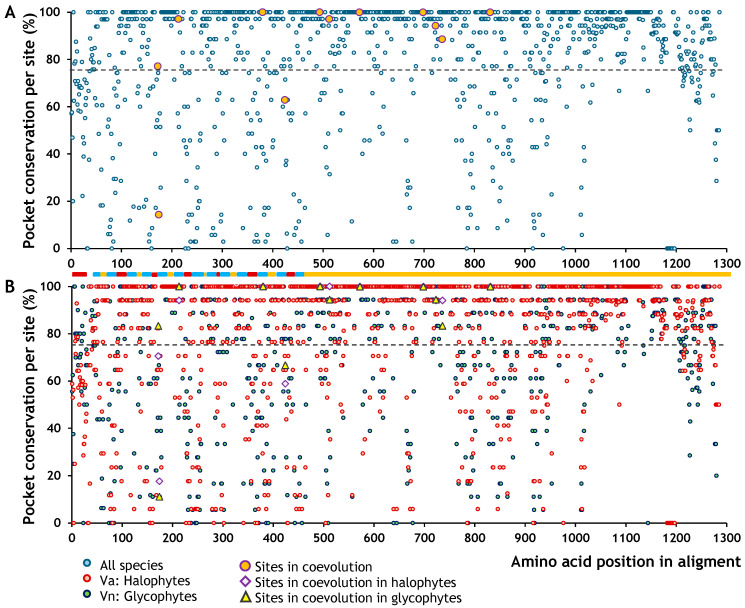
Mapping the site-specific conservation of binding pockets in SOS1. (**A**) Conservation map of binding pockets across all species. Coevolving sites are highlighted with orange circles. (**B**) Conservation map of binding pockets in halophytes and glycophytes. Yellow triangles indicate coevolving sites in glycophytes, and purple diamonds represent coevolving sites in halophytes. The dotted line marks the 75% conservation threshold for pocket presence. Functional domains are colour-coded: C (orange) corresponds to the cytoplasmic topological domain; E (dark red) represents the extracellular domain; and H (blue) marks the transmembrane α-helical segments.

Each amino acid in SOS1 may or may not be associated with structural regions that form binding pockets. Moreover, the conformation of these pockets can vary between species or even be absent altogether. A pocket is considered highly conserved when the same amino acid position forms part of a pocket in many species. This information allowed us to generate a map showing the probability of each SOS1 residue being part of a pocket. We found that, overall, the probability of a residue being part of a pocket—regardless of taxonomic group (i.e., 100% conservation)—was 34% (Table 5, Figure 4). However, this value rose above 40% when halophytes and non-halophytes were analysed separately. Qualitatively, the binding pockets appeared to be more conserved in the cytoplasmic regions of the protein (Figure 4).

**Figure 5:**
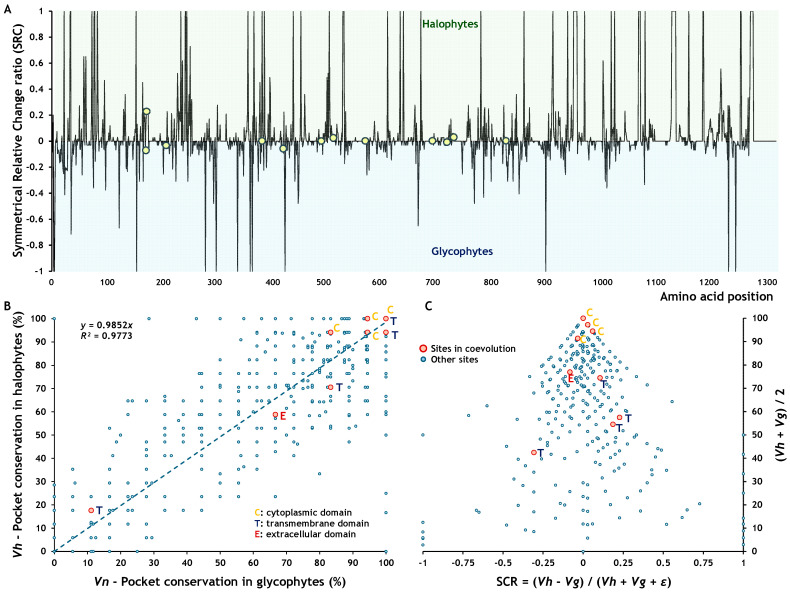
Conservation of molecular structure at coevolving sites in SOS1. (**A**) Symmetric Relative Change ratio (SRC), indicating whether a pocket is more conserved in halophytes (SRC > 0) or in glycophytes (SRC < 0); values near zero reflect similar pocket conservation in both plant groups. (**B**) Linear regression of pocket conservation values in halophytes versus glycophytes. (**C**) SRC plotted against the average conservation (*Vh* + *Vg*)/2, where sites with SRC = 0 and high mean values correspond to highly conserved pockets. Red circles indicate the positions of coevolving sites in SOS1.

Most coevolving sites corresponded to residues with high binding pocket conservation (greater than 75%), including five fully conserved across all species (Figure 4A). Only five coevolving sites were associated with residues showing pocket conservation values below 75%. A distinct pattern was also observed in pocket conservation between halophytes and glycophytes (Figure 4B). This difference became more apparent when we calculated the symmetric relative change (SRC) between the two groups. Positive SRC values indicate higher pocket conservation in halophytes than glycophytes, whereas negative values indicate the opposite. SRC values near zero reflect similar conservation levels between the two groups, regardless of the absolute conservation value. Most coevolving sites were associated with SRC values close to zero, except for one site (Figure 5A), suggesting that their conservation levels were similar in halophytes and glycophytes, although the degree of conservation remained unknown. To evaluate how conserved these coevolving sites were, we performed a linear regression between the pocket conservation values of halophytes and glycophytes (Figure 5B). The relationship showed a strong linear trend (*R*^2^ = 0.9773), indicating that all coevolving sites located in the cytoplasmic domain, and two in the transmembrane domain, belonged to highly conserved pockets. Finally, we plotted SRC against the mean pocket conservation to visualise whether coevolving sites with a zero SRC value were also highly conserved. This analysis confirmed that the sites in the cytoplasmic domain corresponded to residues within highly conserved binding pockets (Figure 5C).

#### 2.4.2. Effects of Amino Acid Residues on NHX1 Topology

**Figure 6:**
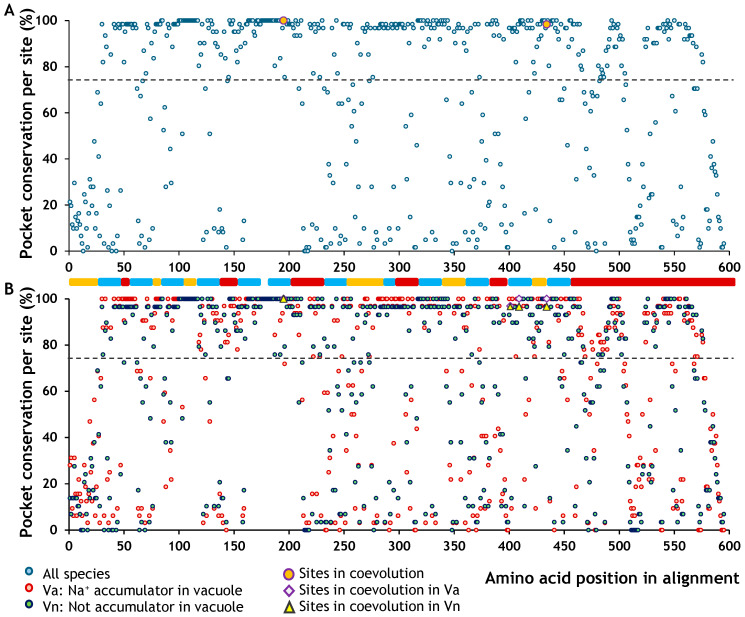
Mapping the site-specific conservation of binding pockets in NHX1. (**A**) Conservation map of binding pockets across all species. Coevolving sites are highlighted with orange circles. (**B**) Conservation map of binding pockets in plants that accumulate Na^+^ in the vacuole and plants that do not. Purple triangles indicate coevolving sites in plants that accumulate Na^+^ in the vacuole, and yellow diamonds represent coevolving sites in plants that do not accumulate Na^+^ in the vacuole. The dotted line marks the 75% conservation threshold for pocket presence. Functional domains are colour-coded: C (orange) corresponds to the cytoplasmic topological domain; E (dark red) represents the vacuolar domain; and H (blue) marks the transmembrane α-helical segments. The four coevolving sites exhibited SRC ratio values below 0.018 (Figure 7A), indicating that their pocket conservation is highly similar in plants accumulating Na^+^ in the vacuole (CRS > 0) and non-accumulating plants. Regarding the overall level of conservation across both groups, the linear regression revealed a strong trend (*R*^2^ = 0.986), with all values clustering above 96% (Figure 7B). Moreover, the last test confirmed that all pairs of coevolving sites belong to highly conserved pocket residues (Figure 7C).

Only 14% of the residues belonged to a binding pocket conserved across all studied species, that is, 100% conservation, in NHX1 (Table 6). When analysed separately, plants that accumulate Na^+^ in the vacuole showed an increase in this value to over 30% (Table 6). The coevolving sites are located in transmembrane regions and correspond to residues with very high pocket conservation, above 96.7% (Figure 6A). In the plants that accumulate Na^+^ in the vacuole, three of the four coevolving sites were fully conserved (100%) and the fourth showed 96.88% conservation. In contrast plants that do not accumulate Na^+^ in the vacuole had three sites with 96.55% conservation and one with 100% (Figure 6B).

**Figure 7:**
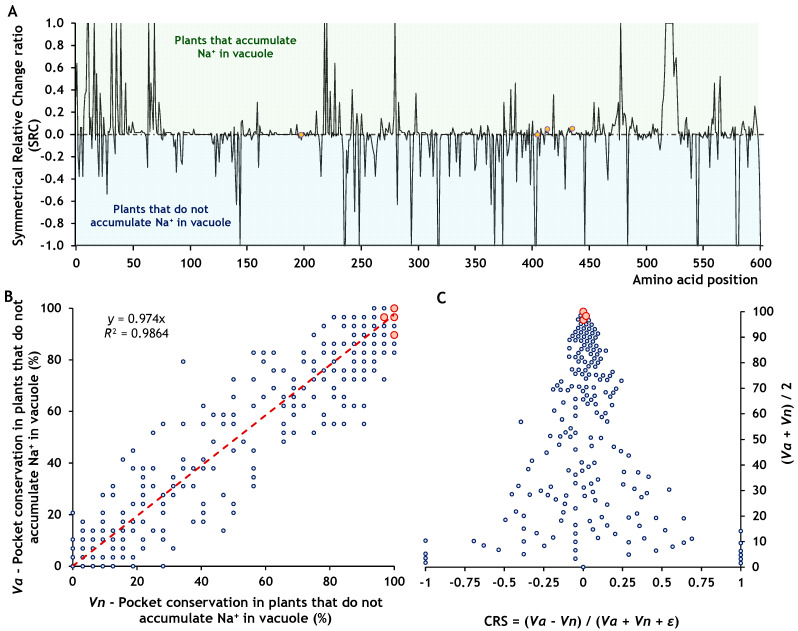
Conservation of molecular structure at coevolving sites in NHX1. (**A**) Symmetric Relative Change ratio (SRC), indicating whether a pocket is more conserved in plants that accumulate Na^+^ in the vacuole (SRC > 0) or in plants that do not (SRC < 0); values near zero reflect similar pocket conservation in both plant groups. (**B**) Linear regression of pocket conservation values in plants accumulating Na^+^ in the vacuole versus those that do not. (**C**) SRC plotted against the average conservation (*Vh* + *Vn*)/2, where sites with SRC = 0 and high mean values correspond to highly conserved pockets. Red circles indicate the positions of coevolving sites in NHX1.

### 2.5. Overall Effects on Function and Topology

**Figure 8:**
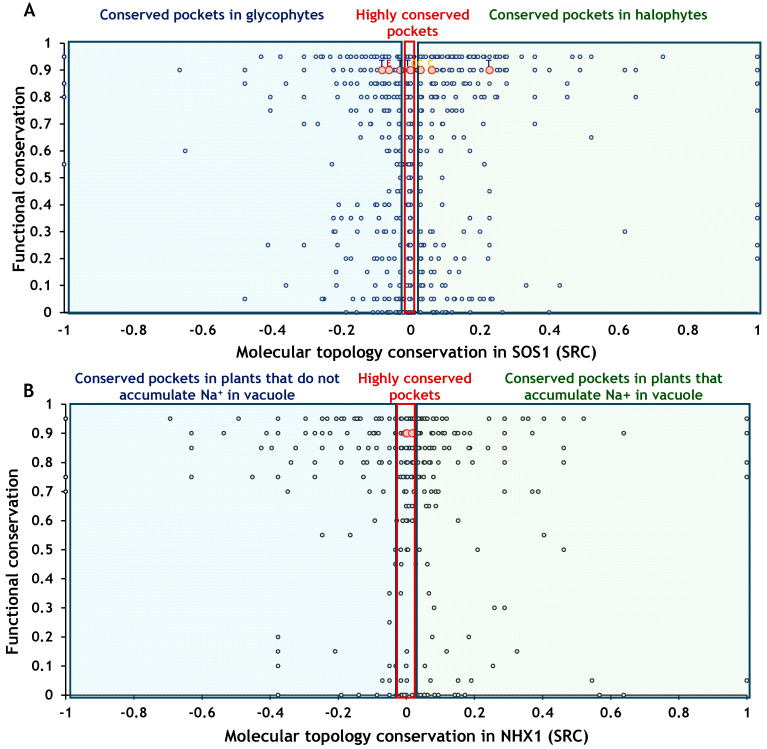
Relationship between residue function and topological context in SOS1 and NHX1 proteins. The *Y*-axis represents the predicted intolerance to mutation for each residue, with values closer to 1 indicating a greater potential impact on protein function. The *X*-axis shows the conservation level of the binding pocket associated with each residue. Red circles indicate the positions of coevolving sites within the protein. (**A**) In SOS1, each residue is classified into one of three categories: pockets conserved in glycophytes, pockets conserved in halophytes, and pockets highly conserved regardless of plant group. (**B**) In NHX1, each residue is classified into one of three categories: pockets conserved in plants that accumulate Na^+^ in vacuoles, pockets conserved in plants that do not accumulate Na^+^ in vacuoles, and pockets highly conserved regardless of plant group.

The combined analysis of functional constraint and topological conservation for amino acid residues in SOS1 and NHX1 revealed an hourglass-shaped distribution (Figure 8A,B). All identified coevolving residue pairs were located at sites with high predicted functional constraint (deleteriousness > 0.9). In SOS1, these coevolving sites exhibited a broader range of structural conservation, with some residues falling into highly conserved pockets and others showing divergence between halophytes and glycophytes. In contrast, NHX1 coevolving sites were located within structurally conserved pockets, irrespective of the Na^+^ accumulation strategy.

## 3. Discussion

This study presents the first identification of intra- and intermolecular coevolving site pairs in the genes *NHX1* and *SOS1*. In both genes, the coevolving sites are associated with residues exhibiting high structural and functional conservation levels, which reinforces the biological relevance of the sites detected through our in silico analysis. All eight coevolving site pairs identified are linked to functional deleteriousness scores of 0.9, indicating that these residues remain under intense selective pressure, yet retain a limited potential to mutate and coevolve.

In SOS1, a key protein in the salt tolerance pathway in plants, we identified three coevolving site pairs located within the cytoplasmic domain. These site pairs correspond to residues highly conserved functionally and structurally, suggesting strong selective constraints. Although the precise molecular mechanisms regulating SOS1 activity remain incompletely understood, the cytoplasmic domain, unique among Na^+^/H^+^ antiporters, has been highlighted as a regulatory hub with a pivotal role in modulating transporter function [41]. One of the coevolving site pairs we identified comprises residues F479 and E703. Previous structural studies have shown that F479 contributes to the interaction between two of the eight α-helices (HC2 and HC7) that form the cytoplasmic domain, stabilising its configuration [42]. E703, on the other hand, located in helix HC8, was found to be part of a functional pocket that interacts with HC1 and plays a role in a structural blocking mechanism, which maintains SOS1 in an inactive conformation by hindering Na^+^ transport [42]. Our coevolutionary analysis suggests that F479 and E703 may have evolved in a coordinated manner, possibly to maintain or fine-tune the delicate balance between active and inactive conformations. Although site-directed mutagenesis of E703 alone did not produce a strong phenotypic effect in isolation, this does not necessarily imply a lack of functional relevance, particularly given its proposed structural role. It remains possible that substitutions at F479 could influence E703 indirectly, potentially altering the conformation of the cytoplasmic domain and its regulatory dynamics. The coevolutionary relationship observed between these residues adds support to their functional significance and may reflect a broader evolutionary tendency to maintain interactions that are critical for protein stability or regulation. Not all coevolving pairs, however, were spatially adjacent in the 3D model. For instance, residues S/G412 and L/I811 lie in the cytoplasmic and extracellular domains, respectively. While they cannot interact directly, their coevolution may reflect indirect constraints, such as compensatory effects on folding or long-range functional coupling between intra- and extracellular regions, which is plausible given the conformational changes required during sodium/proton exchange.

In NHX1, two pairs of coevolving residues were identified within the transmembrane domain. Rombolá-Caldentey et al. [38] reported several highly conserved active sites in NHX1, including N184 and R390, which are spatially close to two of the residues identified in our analysis, T187 and G391. Given this proximity, it is plausible that functional interactions exist between our coevolving residue pairs and the previously characterised active sites. Moreover, the generation of the double mutant N184D–R390K was shown to result in a loss of salt tolerance [38], further supporting the functional relevance of this region. It is also possible that G391 forms part of the active site. In fact, previous work has estimated that approximately 15% of active sites coevolve with other positions, with 22% of those coevolutionary relationships occurring between two active sites, where many coevolving residues tend to be located in close proximity to functionally important active sites [43]. While further functional validation is required, the observed coevolutionary patterns provide supporting evidence that these transmembrane residues may contribute to the functional architecture of NHX1.

Our analyses show that 67% of the residues in SOS1 and 78% in NHX1 are associated with a high predicted functional impact when mutated. All coevolving residues identified in both genes fall within positions under strong selective constraint (deleteriousness ≥ 0.9). Interestingly, none of these sites reached the maximum deleteriousness score of 1.0, implying that while these residues are highly constrained, they may retain enough evolutionary flexibility to support compensatory changes. This suggests the existence of a narrow but critical window for coevolution to operate without compromising essential protein functions.

The topological conservation of pocket-associated sites reveals that coevolving residues are generally located within functional pockets across most species analysed. Although binding pockets are traditionally associated with enzymes, similar structural features in membrane proteins can be functionally relevant. In SOS1, a transmembrane pocket may coordinate or translocate ions, while pockets in the cytoplasmic tail, often disordered, could serve as docking sites for regulatory proteins. In SOS1, the residues with the lowest levels of structural conservation, both in halophytes and glycophytes, are located in the transmembrane domain, not in the cytoplasmic domain, which constitutes an essential region of the protein. This suggests that, while they are not essential for the core transport function, they may provide functional flexibility, allowing adjustments in ion selectivity or transport efficiency. Since the cytoplasmic domain is directly involved in Na^+^ sensing and transport, the consistency between conserved pocket topology and predicted functional constraint aligns well with its proposed role [44].

We identified residues associated with pockets that are highly conserved exclusively in halophytes or glycophytes for SOS1, and likewise in Na^+^ accumulating versus non-accumulating plants for NHX1. These group-specific patterns suggest distinct selective pressures shaped by ecological history and potentially divergent activity profiles of the proteins. Since molecular pockets mediate key interactions with other molecular partners [45], such differences may reflect functional specialisation. However, detecting coevolutionary signals at highly conserved sites is inherently challenging, as the subtle changes involved are often difficult to detect [30]. Residues showing strong conservation only within specific plant groups might represent evolutionary specialisations contributing to physiological divergence. The consistent presence of pocket-associated residues among all coevolving sites in NHX1 further supports the idea that these positions may form part of the protein’s active site, where coevolution is essential for maintaining structural and functional integrity [46].

The analysis of function and topology found a peculiar hourglass-shaped residue distribution. The waist of the hourglass, where SRC values cluster around zero, suggests that residues under moderate functional constraint tend to occur in structurally conserved regions, potentially reflecting general topological roles maintained across taxa. Thus, the distribution of NHX1 sites appears skewed towards higher functional constraint, whereas SOS1 displays a more even spread. This pattern may indicate that NHX1 is under tighter functional constraints, potentially allowing less flexibility in adaptive change than SOS1.

The possibility of intermolecular coevolution between SOS1 and NHX1—two proteins that do not interact directly—has been raised based on experimental evidence showing that both genes are upregulated in response to salt exposure and may act in coordination to regulate Na^+^ allocation across plant tissues [47,48]. Similar cases have been described in other systems, where compensatory evolution reflects functional rather than structural interactions [49]. While this functional convergence is consistent with a scenario of adaptive co-regulation, the available sequence data were insufficient to provide convincing molecular evidence for intermolecular coevolution. Thus, our observation may simply reflect strong selective pressures, as both proteins are highly conserved membrane-bound antiporters. Therefore, our results should be considered exploratory but valuable as a basis for future studies aimed at disentangling the structural and functional mechanisms underlying the coordinated evolution of SOS1 and NHX1, either through intermolecular coevolution or as a consequence of strong selective pressures acting on conserved residues.

To conclude, our findings offer new insights into the coevolutionary dynamics and selective constraints acting on *SOS1* and *NHX1*, two central components of plant salinity tolerance. The combination of functional and structural conservation at coevolving sites underscores their potential importance in protein regulation and adaptation. This study provides a foundation for future experimental work and comparative analyses across broader plant lineages. Finally, identifying conserved and lineage-specific sites offers a molecular framework for developing biotechnological strategies to improve salinity tolerance—an essential target for crop adaptation to climate change.

## 4. Materials and Methods

**Figure 9:**
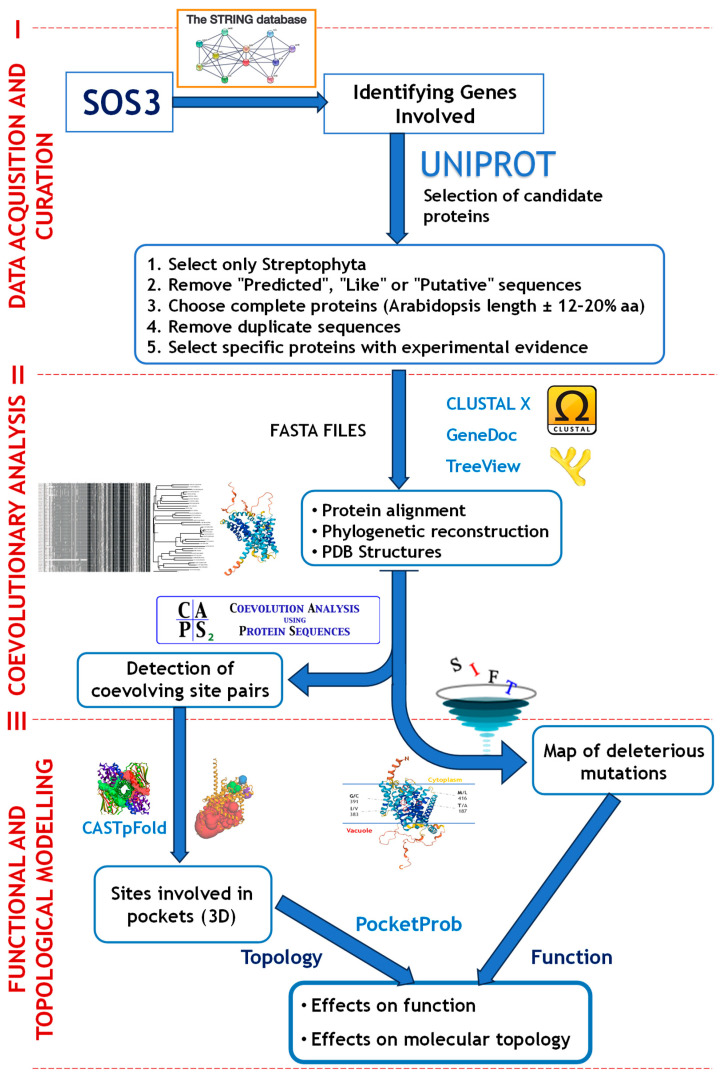
Methodological workflow for the coevolutionary analysis of plant ion transport proteins. The methodology followed consists of three main steps: (I) data acquisition, sequence curation, and gene selection; (II) coevolutionary analysis and identification of coevolving sites; and (III) topological and functional analysis of the amino acid sites involved.

The methodological approach in this study comprised three main phases: data acquisition and curation of ion transport-related protein sequences; coevolutionary analysis to detect patterns of intra- and intermolecular residue covariation; and computational modelling to evaluate the functional and structural impact of coevolving amino acid substitutions (Figure 9).

### 4.1. Data Acquisition and Sequence Curation

SOS3, also known as CBL4, functions as the initiating protein in the activation of the SOS signalling pathway, mediating the Ca^2+^-dependent activation of SOS2 and SOS1. For this reason, SOS3 was used as the entry point in the STRING database [50], version 12 (https://string-db.org/; accessed 8 November 2024), was used to retrieve the core set of proteins associated with the SOS pathway. This choice reflects the central regulatory role of SOS3, rather than its sequence conservation. The model organism used for the analysis was *Arabidopsis thaliana*. The interaction confidence threshold was set to the highest level (0.900). Additionally, a maximum of 50 proteins was included in the first interaction shell and up to five in the second shell.

Using the list of proteins identified through STRING, the UniProt database [51] (https://www.uniprot.org; accessed 8 November 2024) was used to quantify the sequence diversity available for each protein. To limit the dataset to plant proteins, the taxonomic group “Streptophyta” was selected (Figure 9). Data for each protein were downloaded in Excel format and filtered to exclude entries corresponding to partial or putative sequences, specifically, those containing the keywords “predicted”, “like”, or “putative”. Thus, sequences with transcript- and/or protein-level evidence were prioritised, and we reduced the likelihood of including automatically annotated or hypothetical sequences without experimental validation. Sequence completeness was assessed by using the *Arabidopsis thaliana* sequence as a reference. Only those sequences whose lengths fell within ±12–20% of the *A. thaliana* reference were retained to exclude partial, truncated, or non-functional duplicates, even if this strategy entailed discarding some potentially complete variants. In addition, only sequences supported by both protein- and transcript-level evidence were selected. Duplicate entries within the same species, as well as any remaining partial sequences, were removed. The resulting curated list of accessions was used to download amino acid sequences in FASTA format (Figure 9).

While coevolutionary analyses generally benefit from large datasets, with optimal performance reported at around 400 sequences [52], or between one and five times the number of aligned residues [53], recent studies have shown that meaningful coevolutionary signals can be detected with as few as 20 sequences [54]. Based on this finding, a conservative minimum threshold of 35 high-quality sequences was established for this study. This criterion was met only by two proteins, SOS1 and NHX1 (with 62 and 36 sequences, respectively; Supplementary Materials, Tables S1 and S2), which were therefore selected for analysis. For these proteins, both amino acid sequences and experimentally resolved or predicted structures in PDB format were retrieved for further analysis.

### 4.2. Intra- and Intermolecular Coevolutionary Analysis

The detection of coevolutionary interactions requires careful pre-processing of protein sequences. Amino acid sequences in FASTA format, beginning with the *A. thaliana* sequence (Figure 9), were first aligned using ClustalX [55] version 2.0. The resulting alignment file (.aln) was then manually refined using GeneDoc [56] version 2.7 to correct any misalignments. The curated alignment was then re-imported into ClustalX to generate a phylogenetic tree using the neighbour-joining method with 1000 bootstrap replicates (.phb), which was visualised with TreeView [57] version 1.6.6. Using the curated sequence alignments, the reconstructed phylogeny, and the PDB structure, potential intra- and intermolecular coevolutionary interactions for both genes were assessed (Figure 9).

Intramolecular coevolutionary analyses of amino acid sequences from different species were performed using CAPS [58] version 2.0 for SOS1 and NHX1. Since CAPS is not natively compatible with Windows systems, a virtual machine running Ubuntu 24.04.2 LTS was set up to execute the software. CAPS requires the aligned sequence file and the PDB structure corresponding to the *A. thaliana* reference. Analyses were initially conducted using default parameters, including a *p*-value threshold of 0.001. To increase stringency and minimise false positives, albeit with an increased risk of Type II error, the analyses were repeated with a more conservative *p*-value threshold of 0.0001.

For the intermolecular coevolutionary analysis, the procedure closely followed that used for intramolecular analysis, with the difference that CAPS was applied to the pairwise sequence alignments of the two genes involved. Calculations were performed analogously to the intramolecular approach. No significant results were obtained at a threshold of *p*-value < 0.0001, so a less stringent threshold of *p*-value 0.001 was used. Given the limited number of shared species with available sequences for both genes, this analysis is considered exploratory.

### 4.3. Effects of Amino Acid Residues on SOS1 and NHX1 Function

The potential functional impact of mutations at coevolving sites (Figure 9) was evaluated using the SIFT Aligned Sequences tool [59] (https://sift.bii.a-star.edu.sg/, accessed 2 June 2025). This software predicts whether an amino acid substitution will likely affect protein function by analysing sequence homology and the physicochemical properties of amino acids. Aligned amino acid sequences were uploaded using default settings. For each position, SIFT identified which amino acid substitutions were predicted to be functionally intolerant. To quantify the potential deleteriousness at each site, we calculated a score by dividing the number of intolerant substitutions by 20 (i.e., the total number of possible amino acid replacements). These scores were then used to generate a probability map in Excel, highlighting residues predicted to be functionally critical versus those more tolerant to variation. Finally, we examined whether the identified coevolving residue pairs were located in positions predicted to be intolerant, thus assessing their potential functional relevance.

### 4.4. Effects of Amino Acid Residues on SOS1 and NHX1 Topology

An additional approach to evaluate potential selective pressures driving coevolutionary dynamics involves assessing the impact of amino acid substitutions on the topological structure of a protein. Due to their distinct physicochemical properties, different amino acids can induce conformational changes, ranging from subtle to substantial, that alter the protein’s three-dimensional structure. Such structural modifications may impair protein function, for instance, by disrupting interactions with Na^+^. Thus, an important structural feature of proteins is the presence of concave and often partially enclosed regions formed by the folding of the polypeptide chain, commonly referred to as “pockets.” These pockets are defined by their geometry, depth, and chemical composition, and can play pivotal roles in molecular interactions, such as ligand binding, enzymatic activity, and ion coordination. Thus, analysing the conservation of pockets is key to understanding protein functionality.

To evaluate whether the coevolving residues previously identified in our analysis play a relevant role in protein topology, we examined their presence within structural pockets. We further assessed whether the residues forming these pockets were conserved across the phylogeny, as a proxy for their potential impact on protein stability and function. To do this, all available Protein Data Bank (PDB) files corresponding to the three-dimensional structures of the protein sequences used in the SOS1 and NHX1 alignments (Figure 9) were retrieved. Each structure was individually uploaded into the CASTpFold tool [60] (https://cfold.bme.uic.edu/castpfold/, accessed 6 June 2025), which identifies and characterises surface pockets within protein models. The programme generates a graphical representation of the protein conformation and identifies, measures, and describes the topological properties of protein structures, providing a list of amino acid residues involved in surface pockets, internal cavities, and channels. For each sequence, the software outputs a text file indicating the position of each residue, the corresponding amino acid, and, if applicable, the pocket to which it belongs. One sequence in each dataset, SOS1 and NHX1, lacked an associated PDB file (thus, we analysed 34 sequences for SOS1 and 61 sequences for NHX1).

Manually handling such a large volume of data (61 sequences × 596 residues and 35 sequences × 1328 residues, respectively) would be extremely time-consuming. Once the structural information was available, a C++ programme, named PocketProb version 1.0 (available on request), was developed using Visual Studio 2022 (Microsoft Corporation, 2024) to facilitate the calculation of pocket conservation (Figure 9). PocketProb generated an output file that could be easily imported into Excel, indicating for each species and each position in the original aligned sequence whether the residue corresponded to a pocket (1), did not (0), or was a gap in the alignment (-). This allowed straightforward calculation of the proportion of sequences in which each residue was involved in a pocket, a metric we refer to as pocket conservation. Higher values of pocket conservation (close to or at 100) indicate that a residue is consistently part of a pocket across the phylogeny of the available species.

A potentially interesting aspect to analyse is whether different salt tolerance mechanisms are reflected in differences in the conservation of pockets in SOS1 and NHX1. In the case of SOS1, species were categorised as halophytes or glycophytes. However, for NHX1, it is possible to evaluate whether differences exist between species that cope with salt stress by sequestering Na^+^ into vacuoles, where NHX1 plays a key role, and those that do not use this strategy. To investigate this hypothesis, each species was searched in eHaloph (https://ehaloph.uc.pt/), a database of halophytes and other salt-tolerant plants. We identified 16 and 28 halophytic species for SOS1 and NHX1, respectively. To confirm that the remaining species were not halophytes, targeted literature searches were conducted, leading to an updated list of 17 and 40 halophytic or halotolerant species for SOS1 and NHX1, respectively. For NHX1, a further literature review was conducted to identify which of these species actively store Na^+^ in vacuoles, resulting in a refined list of 32 species. A new calculation of pocket conservation per residue was then performed for each of the two plant groups: for SOS1, halophytes (*n* = 17) and glycophytes (*n* = 18); and for NHX1, plants that accumulate Na^+^ in vacuoles (*n* = 32) and plants that do not accumulate Na^+^ in vacuoles (*n* = 29). To obtain a stable, symmetrical, scaled, and normalised metric of the change in the percentage of pocket conservation between both plant groups, we devised the Symmetrical Relative Change ratio (SRC):

$${SRC}_{SOS1}=\left(\frac{\left(Vh-Vg\right)}{\left(Vh+Vg+\varepsilon \right)}\right);\;{SRC}_{NHX1}=\left(\frac{\left(Va-Vn\right)}{\left(Va+Vn+\varepsilon \right)}\right)$$

where *Vh* is the percentage of pocket conservation for each residue in halophytic species (for SOS1) and *Va* in species that accumulate Na^+^ in vacuoles (for NHX1); *Vg* and V*n* are the percentages of pocket conservation in glycophytes or in plants that do not accumulate Na^+^ in the vacuole, respectively; and ε is a pseudo-count (ε = 10^−6^) used to avoid division by zero when a given site is not conserved in either group (*Vh* = *Vg* = 0 or *Va* = *Vn* = 0, respectively). The metric is symmetrical, meaning that switching the values of halophytes and glycophytes only alters the sign of the result:

$$e.g.,\;\frac{Va-Vg}{Va+Vg+\varepsilon }=-\frac{Vg-Va}{Va+Vg+\varepsilon }$$

It is bounded because SRC ratio values range between −1 and +1, where +1 indicates residues located in pockets that are highly conserved in halophytic species or those that accumulate Na^+^ in vacuoles; −1 indicates residues highly conserved in non-halophytic or non-accumulating species; and 0 indicates equal conservation in both plant groups, regardless of the absolute conservation percentage.

In addition to calculating the SRC ratio, the overall conservation pattern of residues between halophytes (or species that accumulate Na^+^ in vacuoles) and glycophytes (or species that do not accumulate Na^+^ in vacuoles) was assessed using a linear regression between *Vh* or *Va* and *Vg* or *Vn*. However, when the SRC ratio is calculated, it may occur that, for the same residue, pocket conservation in both plant groups is equal, resulting in a SRC ratio value of 0, regardless of whether the conservation level is high or low. To graphically distinguish highly conserved sites from non-conserved ones in the SRC, the average pocket conservation was calculated as follows for SOS1 (and, similarly, for NHX1):

$$Average=\left(\frac{Vh+Vg}{2}\right)$$

### 4.5. Overall Effects on Function and Topology

Coevolving residues may be shaped by selective pressures acting simultaneously at the functional and structural levels. To explore this, we examined the relationship between predicted deleteriousness and pocket conservation per residue. Specifically, deleteriousness scores—representing the likelihood that amino acid substitutions disrupt protein function—were compared with CRS values, which quantify shifts in structural conservation of pockets between plant groups. This analysis aimed to determine whether sites under functional constraint also exhibit structural conservation, providing evidence of coordinated selective pressures.

## 5. Conclusions

This study provides the first coevolutionary perspective on the SOS interaction network, revealing conserved molecular relationships in SOS1 and NHX1 that are critical for ion homeostasis. The identification of coevolving residues in domains essential for Na^+^ transport highlights functionally relevant sites conserved across both plant lineages and strategies to cope with salinity, such as Na^+^ accumulation in vacuoles. Importantly, our results suggest clear experimental strategies to validate these predictions, such as comparing single and double mutants in SOS1 or NHX1 backgrounds and assaying Na^+^/H^+^ transport activity under salt stress. Such approaches would allow testing whether paired mutations reveal compensatory effects, thereby confirming the functional connection between coevolving residues. These insights contribute to a deeper understanding of the molecular basis of salt tolerance and offer potential targets for future biotechnological applications to improve crop resilience under saline conditions.

## Figures and Tables

**Table 1:** Proteins interacting with SOS3 and their corresponding UniProt sequences. For all proteins reported to interact with SOS3 according to the STRING database, homologous sequences were retrieved and filtered to retain only complete, non-redundant sequences from plant species. Partial, duplicated, or non-plant sequences were excluded from the analysis. Based on the final number of validated sequences, only SOS1 and NHX1 (in bold) met the criteria for downstream analyses.

Protein	Synonyms	Number of Sequences	Valid Sequences	Function
**Direct interactions with SOS3 (CBL4)**				
SOS3	CBL4	37	15	Calcineurin B-like 4 protein
AKT1	At2g26650, F18A8.2	4776	10	Potassium channel AKT1
CBL10	At4g33000, F26P21_120, SCABP8	40	10	Calcineurin B-like 10 protein
CIPK1	PKS13, SnRK3.16, At3g17510, MKP6.6	39	6	Protein 1 CBL serine/threonine kinase
CIPK11	PKS5, SIP4, SnRK3.22, At2g30360, T9D9.17	46	12	Protein 11 CBL serine/threonine kinase
CIPK14	PKS24, SnRK3.15, SR1, At5g01820, T20L15.90	55	14	Protein 14 CBL serine/threonine kinase
SOS2	CIPK24, SnRK3.11, At5g35410, F6I13.1, K21B8.3	47	16	Protein 24 CBL Serine/Threonine-Kinase Interaction
CIPK6	PKS4, SIP3, SnRK3.14, At4g30960, F6I18.130	43	8	Protein 6 CBL serine/threonine kinase
**NHX1**	**At5g27150, T21B4.60**	**176**	**62**	**Na^+^/H^+^ Antiporter 1**
**SOS1**	**NHX7, At2g01980, F14H20.5**	**356**	**36**	**Na^+^/H^+^ Antiporter 7**
**Second layer of interactions with SOS3 (CBL4)**				
AKT2	AKT3, At4g22200, T10I14.30	620	2	AKT2/3 K^+^ Channel
CBL1	SCABP5, At4g17615, dl4845w, FCAALL.122	65	14	Calcineurin B-like protein 1
CBL2	SCABP1, At5g55990, MDA7.3	42	14	Calcineurin B-like 2 protein
CBL3	SCABP6, At4g26570, T15N24.20	57	13	Calcineurin B-like 3 protein
CBL9	At5g47100, K14A3.5	53	8	Calcineurin B-like 9 protein

**Table 2:** Intramolecular coevolution analysis in SOS1 and NHX1. CAPS identified six pairs of coevolving sites (AA1 and AA2), using an alpha value of 0.0001 and 100 simulations, thereby reducing the likelihood of false positives. For each site, the following information is provided: the residue in *Arabidopsis thaliana* sequences (SOS1: Q9LKW9, NHX1: Q68KI4) that is in coevolution, and, in parentheses, the position of the residue within the alignment; when the Pearson correlation coefficients are one, or close to one, indicate that the evolutionary changes at the two positions are perfectly correlated across the branches of the phylogenetic tree; bootstrap values; the amino acid pairs involved (AA1–AA2); the percentage of sequences (based in 36 sequences for SOS1 and 62 for NHX1) in which the pair occurs; and the phylogenetic groups in which it is found.

Position AA1	Position AA2	Corr.	Boots	AA Pairs	%	Taxonomic Groups
**SOS1**						
162 (172)	164 (174)	0.95	1.00	L-L F-F	86.5 13.5	All taxa except: *Aegilops Triticum Group*
203 (213)	368 (380)	1.00	1.00	T-V I-A	94.6 5.4	All taxa except: Caryophyllales: *Bassia*, *Salicornia*
412 (424)	811 (831)	1.00	1.00	S-L G-I	94.6 5.4	All taxa except: Malpighiales: *Ricinus*, Fabales: *Glycine*
479 (493)	703 (723)	1.00	1.00	F-E Y-Q	94.6 5.4	All taxa except: Caryophyllales: *Fagopyrum*
497 (512)	716 (736)	1.00	1.00	K-R E-K	94.6 5.4	All taxa except: Brassicales: *Arabidopsis*, *Eutrema*
554 (572)	678 (698)	1.00	1.00	Q-D E-G	94.6 5.4	All taxa except: Brassicales: *Arabidopsis*, *Eutrema*
**NHX1**						
187 (195)	416 (434)	1.00	0.96	T-M A-L	96.77 3.23	All taxa except: Saxifragales: *Paeonia* sp. Gentianales: *Gentiana* sp.
383 (401)	391 (409)	1.00	0.87	I-G V-C	96.77 3.23	All taxa except: Saxifragales: *Paeonia* sp. Fabales: *Robinia* sp.

**Table 3:** Intermolecular coevolution analysis between NHX1 and SOS1 sites. Pairs of amino acid positions in the SOS1 and NHX1 genes showing evidence of potential coevolution were identified. For each pair: the residues in *Arabidopsis thaliana* sequences (SOS1: Q9LKW9, NHX1: Q68KI4) that were found under coevolution and, in parentheses, the position of the residues within the alignment are shown. Pearson correlation coefficients and bootstrap values are also provided. The analysis was performed on the only nine species for which sequences of both proteins were available and, thus, the results are mainly exploratory. A *p*-value threshold of 0.001 was used to assess interspecific coevolution.

Position SOS1	Position NHX1	Correlation	Bootstrap
1043 (1087)	69 (81)	0.526	0.880
31 (38)	75 (87)	0.606	0.954
217 (224)	456 (472)	0.0378	0.826
257 (264)	456 (472)	0.023	0.807
384 (393)	456 (472)	0.507	0.795
911 (929)	456 (472)	0.0575	0.851
991 (1028)	456 (472)	0.0417	0.875

**Table 4:** Functional importance of SOS1 and NHX1 residues based on amino acid substitution intolerance scores. For each protein, the number of residues falling within different ranges of intolerance to amino acid substitution was determined using SIFT (https://sift.bii.a-star.edu.sg/, accessed 2 June 2025). The analysis evaluated the tolerance of each original amino acid to substitution by all other possible amino acids. Percentages were calculated relative to the total number of amino acids in SOS1 (1146) and NHX1 (538). #: number.

	SOS1		NHX1	
Inactivation Ranks	# Residues	% Sites	# Residues	% Sites
**0.00**	58	5.1	23	4.3
**0.00 to 0.25**	130	11.3	25	4.6
**0.25 to 0.50**	64	5.6	18	3.3
**0.50 to 0.75**	125	10.9	53	9.9
**0.75 to 0.99**	769	67.1	419	77.9
**1.00**	0	0.0	0	0.0

**Table 5:** Conservation of binding pockets in SOS1 across all 35 species, halophytes (*N* = 17) and glycophytes (*N* = 18). The number of amino acids falling within each conservation range is shown for the binding pockets of SOS1, calculated across all plant species (Total), as well as separately for halophytes (H) and glycophytes (G). The corresponding percentages for each group are also provided.

Binding Pocket Conservation Range	Total	G	H	Total (%)	G (%)	H (%)
**0**	24	29	32	1.87	2.37	2.50
**0 to 25%**	80	68	79	6.22	5.55	6.17
**25 to 50%**	87	87	72	6.77	7.10	5.63
**50 to 75%**	156	136	154	12.13	11.09	12.03
**75 to 99%**	497	324	415	38.65	26.43	32.42
**100%**	442	582	528	34.37	47.47	41.25

**Table 6:** Pocket conservation in NHX1 across all 61 species, and separately for plants that accumulate Na^+^ in the vacuole (PAV, *N* = 32) and plants that do not accumulate Na^+^ in the vacuole (PnAV, *N* = 29) plants. The number of amino acids within each pocket conservation range is shown for all species (total), and for each plant group (PAV and PnAV), along with their respective percentages.

Binding Pocket Conservation Range	Total	PAV	PnAV	Total (%)	PAV (%)	PnAV (%)
**0**	8	29	27	1.34	4.87	4.54
**0 to 25%**	132	114	119	22.18	19.16	20.00
**25 to 50%**	47	51	39	7.90	8.57	6.55
**50 to 75%**	56	48	60	9.41	8.07	10.08
**75 to 99%**	267	167	243	44.87	28.07	40.84
**100%**	85	186	107	14.29	31.26	17.98

## Data Availability

The original contributions presented in this study are included in the article/Supplementary Materials. Further inquiries can be directed to the corresponding author.

## Supplementary Materials

The following supporting information can be downloaded at: https://www.mdpi.com/article/10.3390/ijms26199276/s1.
