# Supplementary material for: Coevolutionary Patterns in SOS1 and NHX1: Insights into Plant Ion Homeostasis Proteins
Source: Int J Mol Sci. 2025 Sep 23;26(19):9276. doi: 10.3390/ijms26199276 (PMC12525166; doi:10.3390/ijms26199276)
Supplement: Supplementary file 1 [file ijms-26-09276-s001.zip › ijms-3854048-supplementary.pdf]

Supplementary Table 1. SOS1 sequences. For each of the 36 UniProt IDs we provide the taxonomic information and whether the species is considered halophyte.

UniProt ID	Clade	Order	Family	Species	Halophyte
A0A3G9CN77	Eudicots	Asterales	Asteraceae	*Chrysanthemum crassum*	
M4VPF7	Eudicots	Asterales	Asteraceae	*Helianthus tuberosus*	Yes
A0A7M1VF20	Eudicots	Asterales	Asteraceae	*Karelinia caspia*	Yes
Q9LKW9	Eudicots	Brassicales	Brassicaceae	*Arabidopsis thaliana*	
A2TJU4	Eudicots	Brassicales	Brassicaceae	*Eutrema halophilum*	Yes
A2TJU5	Eudicots	Caryophyllales	Aizoaceae	*Mesembryanthemum crystallinum*	Yes
K7X0C8	Eudicots	Caryophyllales	Aizoaceae	*Sesuvium portulacastrum*	Yes
A0A1S7IYZ3	Eudicots	Caryophyllales	Amaranthaceae	*Bassia scoparia*	Yes
A0A140DP51	Eudicots	Caryophyllales	Amaranthaceae	*Halogeton glomeratus*	Yes
W6EIW6	Eudicots	Caryophyllales	Amaranthaceae	*Suaeda salsa*	Yes
A0A0D6DQP6	Eudicots	Caryophyllales	Amaranthaceae	*Salicornia dolichostachya*	Yes
B3VN42	Eudicots	Caryophyllales	Plumbaginaceae	*Limonium gmelinii*	Yes
A0A455JTA9	Eudicots	Caryophyllales	Polygonaceae	*Fagopyrum esculentum*	
A0A2L2FKX5	Eudicots	Caryophyllales	Polygonaceae	*Fagopyrum tataricum*	
A0A067Y2L1	Eudicots	Caryophyllales	Tamaricaeae	*Reaumuria trigyna*	Yes
H9DVC6	Eudicots	Cucurbitales	Cucurbitaceae	*Cucumis sativus*	
H9CDQ2	Eudicots	Fabales	Fabaceae	*Glycine max*	
A0A1L6K365	Eudicots	Malpighiales	Euphorbiaceae	*Ricinus communis*	
F4YIB1	Eudicots	Malpighiales	Rhizophoraceae	*Bruguiera gymnorhiza*	Yes
Q19N58	Eudicots	Malpighiales	Salicacea	*Populus euphratica*	Yes
A0A2L0W0A5	Eudicots	Malpighiales	Salicacea	*Populus ilicifolia*	
U5G5K6	Eudicots	Malpighiales	Salicacea	*Populus trichocarpa*	
A0A165UJW2	Eudicots	Malvales	Malvaceae	*Gossypium hirsutum*	
A0A097DC46	Eudicots	Malvales	Malvaceae	*Kosteletzkya pentacarpos*	Yes
F2Q9A0	Monocots	Poales	Poaceae	*Aegilops speltoides*	
F2Q9A1	Monocots	Poales	Poaceae	*Aegilops tauschii*	
G9JV83	Monocots	Poales	Poaceae	*Aeluropus littoralis*	Yes
V9HXA3	Monocots	Poales	Poaceae	*Distichlis spicata*	Yes
L0HQL4	Monocots	Poales	Poaceae	*Indosasa sinica*	
A0A6F8F8Q2	Monocots	Poales	Poaceae	*Oryza sativa*	
I0JTU6	Monocots	Poales	Poaceae	*Triticum aestivum*	
F2Q999	Monocots	Poales	Poaceae	*Triticum monococcum*	
B2CWA1	Monocots	Poales	Poaceae	*Triticum turgidum*	
A0A067Y3N6	Eudicots	Sapindanales	Nitrariaceae	*Nitraria tangutorum*	Yes
A0A411P2P9	Eudicots	Solanales	Solanaceae	*Lycium ruthenicum*	Yes
D0V1M4	Eudicots	Vitales	Vitaceae	*Vitis vinifera*	

Supplementary Table 1. NHX1 sequences. For each of the 62 UniProt IDs we provide the taxonomic information and whether the species accumulates Na^+ in vacuole (VA).

UniProt ID	Clade	Order	Family	Species	Va
A0A0U3BIP9	Monocots	Asparagales	Iridaceae	*Iris halophila*	Yes
A3RFZ0	Eudicots	Asterales	Asteraceae	*Chrysanthemum morifolium*	Yes
A0A0E3DB78	Eudicots	Asterales	Asteraceae	*Cichorium intybus*	
A1Z1Q2	Eudicots	Asterales	Asteraceae	*Helianthus tuberosus*	Yes
Q68KI4	Eudicots	Brassicales	Brassicaceae	*Arabidopsis thaliana*	
F2YPM9	Eudicots	Brassicales	Brassicaceae	*Olimarabidopsis pumila*	Yes
A6Q0F7	Eudicots	Caryophyllales	Aizoaceae	*Mesembryanthemum crystallinum*	Yes
H9DVC8	Eudicots	Caryophyllales	Aizoaceae	*Sesuvium portulacastrum*	Yes
Q6A4M3	Eudicots	Caryophyllales	Aizoaceae	*Tetragonia tetragonoides*	
Q84U80	Eudicots	Caryophyllales	Amaranthaceae	*Atriplex dimorphostegia*	Yes
Q9FZN0	Eudicots	Caryophyllales	Amaranthaceae	*Atriplex gmelinii*	Yes
A0A023VSZ4	Eudicots	Caryophyllales	Amaranthaceae	*Atriplex halimus*	Yes
A0A0A7AAJ1	Eudicots	Caryophyllales	Amaranthaceae	*Atriplex patens*	Yes
A0FH77	Eudicots	Caryophyllales	Amaranthaceae	*Eutrema halophilum*	Yes
E3T2E8	Eudicots	Caryophyllales	Amaranthaceae	*Halostachys caspica*	Yes
Q3Y5G7	Eudicots	Caryophyllales	Amaranthaceae	*Salicornia bigelovii*	Yes
B1PLB6	Eudicots	Caryophyllales	Amaranthaceae	*Salicornia brachiata*	Yes
Q69IH0	Eudicots	Caryophyllales	Amaranthaceae	*Salicornia europaea*	Yes
Q19PX1	Eudicots	Caryophyllales	Amaranthaceae	*Suaeda corniculata*	Yes
A0A023VU83	Eudicots	Caryophyllales	Amaranthaceae	*Suaeda pruinosa*	Yes
Q84MJ8	Eudicots	Caryophyllales	Amaranthaceae	*Suaeda salsa*	Yes
A0A6B7K4X9	Eudicots	Caryophyllales	Cactaceae	*Selenicereus undatus*	
B3VN41	Eudicots	Caryophyllales	Plumbaginaceae	*Limonium gmelinii*	Yes
E2IQH6	Eudicots	Fabales	Fabaceae	*Arachis hypogaea*	Yes
E0AD90	Eudicots	Fabales	Fabaceae	*Cicer arietinum*	
B0FNA7	Eudicots	Fabales	Fabaceae	*Galega orientalis*	
B3VBF0	Eudicots	Fabales	Fabaceae	*Lotus tenuis*	Yes
A7L689	Eudicots	Fabales	Fabaceae	*Robinia pseudoacacia*	Yes
A7Y1B9	Eudicots	Fabales	Fabaceae	*Trifolium repens*	
G3MCE4	Eudicots	Fabales	Fabaceae	*Vigna unguiculata*	
A0A0A7CBI8	Eudicots	Gentianales	Gentianaceae	*Gentiana rigescens*	
A8WE27	Eudicots	Lamiales	Plantaginaceae	*Plantago maritima*	Yes
A0A0F6X1L9	Eudicots	Malpighiales	Euphorbiaceae	*Ricinus communis*	
K9J9R5	Eudicots	Malpighiales	Salicacea	*Populus euphratica*	Yes
Q8GTL9	Eudicots	Malvales	Malvaceae	*Gossypium hirsutum*	
AAY90136	Monocots	Poales	Poaceae	*Cenchrus americanus*	
G3GJZ6	Monocots	Poales	Poaceae	*Diplachne fusca*	Yes
Q58I37	Eudicots	Gentianales	Gentianaceae	*Festuca arundinaceae*	Yes
Q84XT0	Monocots	Poales	Poaceae	*Hordeum brevisubulatum*	Yes
A0A1B1LUU6	Monocots	Poales	Poaceae	*Hordeum vulgare*	Yes
S4SCC5	Monocots	Poales	Poaceae	*Oryza coarctata*	Yes
A0A6F8FR17	Monocots	Poales	Poaceae	*Oryza sativa*	
A0A158VEJ8	Monocots	Poales	Poaceae	*Panicum virgatum*	Yes
D2Y391	Monocots	Poales	Poaceae	*Phyllostachys dulis*	Yes
ACD64982	Monocots	Poales	Poaceae	*Sorghum bicolor*	
Q5I6M7	Monocots	Poales	Poaceae	*Thinopyrum elongatum*	Yes

A3FMN6	Monocots	Poales	Poaceae	*Thinopyrum intermedium*	Yes
Q94BM4	Monocots	Poales	Poaceae	*Triticum aestivum*	
Q84MI0	Monocots	Poales	Poaceae	*Zea mays*	
A9Z1C0	Monocots	Poales	Poaceae	*Zoysia japonica*	Yes
A0A0A1H8F1	Eudicots	Proteales	Nelumbonaceae	*Nelumbo nucifera*	
A0A0S2SXD1	Eudicots	Ranunculales	Papaveraceae	*Eschscholzia californica*	
D3JYS6	Eudicots	Rosales	Rosaceae	*Malus domestica*	
I7BPB4	Eudicots	Rosales	Rosaceae	*Malus zumi*	Yes
M1LCM0	Eudicots	Rosales	Rosaceae	*Rosa rugosa*	Yes
A0A068EUK0	Eudicots	Sapindanales	Nitrariaceae	*Nitraria tangutorum*	Yes
K4JWE3	Eudicots	Saxifragales	Paeoniaceae	*Paeonia lactiflora*	Yes
A7BJ38	Eudicots	Solanales	Convolvulaceae	*Ipomoea tricolor*	Yes
A0A096VPL7	Eudicots	Solanales	Solanaceae	*Nicotiana benthamiana*	
Q93YH2	Eudicots	Solanales	Solanaceae	*Solanum lycopersicum*	
G3M3X2	Eudicots	Solanales	Solanaceae	*Solanum torvum*	Yes
Q4VT46	Eudicots	Vitales	Vitaceae	*Vitis vinifera*	
